# The invasive lobular carcinoma as a prototype luminal A breast cancer: A retrospective cohort study

**DOI:** 10.1186/1471-2407-10-664

**Published:** 2010-12-03

**Authors:** So-Youn Jung, Junsoo Jeong, Seung-Ho Shin, Youngmee Kwon, Eun-A Kim, Kyoung Lan Ko, Kyung Hwan Shin, Keun Seok Lee, In Hae Park, Seeyoun Lee, Seok Won Kim, Han-Sung Kang, Jungsil Ro

**Affiliations:** 1 Center for Breast Cancer, National Cancer Center, Goyang-si, Korea

## Abstract

**Background:**

Although the invasive lobular carcinoma (ILC) is the second most frequent histologic subtype in Western countries, its incidence is much lower in Asia, and its characteristics are less well known.

**Methods:**

We assessed the clinical characteristics and outcomes of 83 Korean patients (2.8%) with ILC for comparison with 2,833 (97.2%) with the invasive ductal carcinoma (IDC), including 1,088 (37.3%) with the luminal A subtype (LA-IDC).

**Results:**

The mean age of all patients was 48.2 years, with no significant differences among the groups. Compared to IDC, ILC showed a larger tumor size (≥T2, 59.8% vs. 38.8%, *P *= 0.001), a lower histologic grade (HG 1/2, 90.4% vs. 64.4%, *P *< 0.001), more frequent estrogen receptor positive (90.4% vs. 64.4%, *P *< 0.001), progesterone receptor positive (71.1% vs. 50.1%, *P *< 0.001) and HER2 negative (97.5% vs. 74.6%, *P *< 0.001) status, and lower Ki-67 expression (10.3% ± 10.6% vs. 20.6% ± 19.8%, *P *< 0.001), as well as being more likely to be of the luminal A subtype (91.4% vs. 51.2%, *P *< 0.001). Six (7.2%) ILC and 359 (12.7%) IDC patients developed disease recurrence, with a median follow-up of 56.4 (range 4.9-136.6) months. The outcome of ILC was close to LA-IDC (HR 0.77 for recurrence, 95% CI 0.31-1.90, *P *= 0.57; HR 0.75 for death, 95% CI 0.18-3.09, *P *= 0.70) and significantly better than for the non-LA-IDC (HR 1.69 for recurrence, 95% CI 1.23-2.33, *P *= 0.001; HR 1.50 for death, 95% CI 0.97-2.33, *P *= 0.07).

**Conclusions:**

ILC, a rare histologic type of breast cancer in Korea, has distinctive clinicopathological characteristics similar to those of LA-IDC.

## Background

The invasive lobular carcinoma (ILC), known to be the second most common histologic subtype of invasive breast cancer following the invasive ductal carcinoma (IDC), constitutes 8-14% of all breast cancers in most Western reports [1-3]. However, in Asia it appears to be very low, accounting for only 2-4% in Korea [4-6] and 1-4% in Japan [7,8].

Previous studies have demonstrated distinctive clinical and biologic characteristics for ILC as compared with IDC. For example, it is more likely to occur in older patients, be larger in size, be estrogen receptor (ER) and progesterone receptor (PgR) positive and have low to absent human epidermal growth factor receptor-2 (HER2) expression [9,10]. Traditionally, both ILC and IDC subtypes have received the same treatment, depending on their clinicopathological characteristics, and the prognosis is reported to be similar [10,11].

Recently, classification of breast cancers by gene expression profiling into particular subtypes has become established [12]. However, in clinical practice, the combination of expression of hormone receptors and HER2 by immunohistochemistry (IHC) is more commonly used to define breast cancers into the luminal A (ER^+ ^or PgR^+^, HER2^-^), luminal B (ER^+ ^or PgR^+^, HER2^+^), HER2-overexpressing (ER^- ^and PgR^-^, HER2^+^), and triple-negative (TNBC: ER^-^, PgR^-^, HER2^-^) subtypes, which demonstrate major differences in clinical outcomes, with the luminal A subtype showing the best prognosis [13,14]. The relative distributions of these four immunohistochemically defined subtypes in the lobular lesions have yet to be established in detail.

The purpose of the current study was to analyze the characteristics of an ILC series and compare the clinical and prognostic parameters with those of general IDC and of the luminal A subtype of IDC (LA-IDC).

## Methods

### Patients

All patients were treated at the National Cancer Center, Korea during the years from 2001 to 2008. A total of 83 consecutive cases diagnosed with pure ILC, including two cases with synchronous bilateral ILC, were enrolled in the study All ILC cases were classic subtype except for one case which was pleomorphic type. This particular case was triple negative by IHC. To compare clinicopathological characteristics and prognoses, 2,833 consecutive patients diagnosed with IDC during the same period were also selected.

### Clinicopathological evaluation

We retrospectively evaluated conventional clinicopathological factors, including treatment modalities (type of operation, use of chemotherapy, hormone therapy, anti-HER2 therapy and radiotherapy) and the IHC results for five biological factors (ER [SP1], Ventana; PgR [1E2], Ventana; HER2 [polyclonal], DAKO; p53 [Bp53-11], Ventana; and Ki-67 [MIB-1], DAKO) using paraffin-embedded tissues according to the reported recommendations for tumor marker prognostic studies (REMARK) [15]. The pathological tumor stage was assessed according to the criteria described in the 6th edition of the American Joint Committee on Cancer (AJCC) staging manual [16]. The tumor grade was determined according to the Scarff-Bloom-Richardson classification modified by Elston and Ellis [17].

A cut-off value of 10% of positively stained nuclei was used to define ER and PgR positivity; HER2 was scored as 0-3+ by a pathologist (Y. Kwon) according to the method recommended for the Dako Hercep Test. Cases with IHC scores of 3+ or 2+ with gene amplification by fluorescence in situ hybridization (FISH) were considered positive for HER2. Cells with positive staining for Ki-67 and p53 were counted and expressed as a percentage. For p53, we scored the lesions as 0-3+ (0, negative; 1+, ≤25%; 2+, 25-50%; 3+, >50%). For the prognosis comparison, low expression was defined as Ki-67 < 20% and p53 ≤ 25% (median values for all evaluated tumors).

For the subgroup analysis, the definition of Luminal A was as follows: positive ER or PgR by IHC, negative HER2 represented by an IHC score of 0 or 1+, or 2+ if not amplified by FISH. The HER2 cases of an IHC score of 2+ but no FISH results were counted as unknowns. The definitions of the other subtypes were as follows: Luminal B, ER or PgR positive and HER2 positive; HER2 overexpressing, low ER and PgR scores but HER2 positive; TNBC, low ER and PgR scores and HER2-negative.

Treatment, including surgery, adjuvant chemo, endocrine or anti-HER2 therapy, and radiotherapy, was applied equally to patients with ILC and IDC, dependent on the clinicopathological characteristics.

### Statistical analysis

The primary endpoints of this study were disease-free survival (DFS) and overall survival (OS). The DFS period was defined as the interval from the date of diagnosis to the date of the first observation of disease recurrence, either loco-regional recurrence or distant metastasis, or the last follow-up date without any evidence of recurrence. Overall survival was calculated from the date of primary breast cancer diagnosis to the date of death or last follow-up.

To compare the clinicopathological characteristics between pairs of groups, we used the Student's t-test and the chi-square test. The DFS and OS rates were calculated using the Kaplan-Meier method, and the groups were compared using the log-rank test. For the multivariate analysis, Cox regression analysis was applied. Statistical analyses were performed using Stata 10.0 for Windows (Stata Corporation Station, TX, USA).

This study protocol was reviewed and approved by the Institutional Review Board of the National Cancer Center (NCCNCS-10-371), Korea, and it complied with the recommendations of the Declaration of Helsinki for biomedical research involving human subjects. The ethical review board supported that informed consent was not required for this study.

## Results

### Patient characteristics

**Figure 1:**
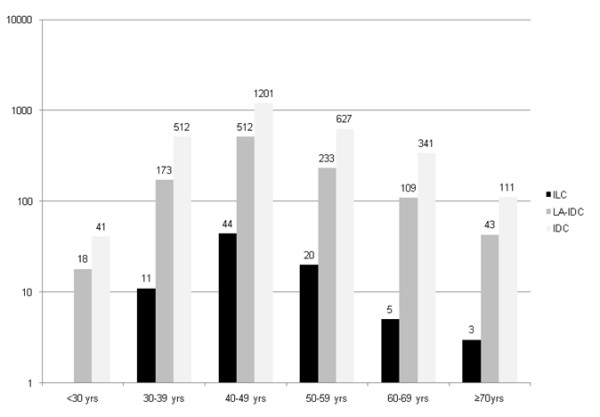
**Age distributions of invasive lobular carcinoma (ILC), invasive ductal carcinoma (IDC), and luminal A subtype of invasive ductal carcinoma (LA-IDC)**.

The clinicopathological characteristics of the 83 ILC patients and 2,833 IDC patients are summarized in Table 1. The mean ages were 48.3 years and 48.2 years, respectively, with no difference in the distributions of age at diagnosis between ILC and IDC (Figure 1). Two patients with mixed lobular and ductal cancers were excluded from the analysis.

**Table 1:** Clinicopathological characteristics of invasive lobular carcinoma, invasive ductal carcinoma, and luminal A subtype

Characteristic		ILC		IDC		*P*	LA-IDC		*P*
		(N = 83)		(N = 2833)			(N = 1088)		
					
		n	%	n	%		n	%	
Mean age (years)		48.3 ± 8.5		48.2 ± 10.5		0.93	47.9 ± 10.2		0.71
pT	T1	33	40.2	1730	61.2	0.001	667	61.4	0.001
	T2	45	54.9	986	34.9		373	34.4	
	T3	4	4.9	89	3.1		37	3.4	
	T4	0	0	23	0.8		9	0.8	
	Unknown	1		5			2		
pN	N0	46	56.1	1639	58.7	0.18	551	51.5	0.25
	N1	24	29.3	763	27.3		333	31.2	
	N2	5	6.1	276	9.9		128	12	
	N3	7	8.5	116	4.2		57	5.3	
	Unknown	1		39			19		
M	M0	83	100	2777	98	0.19	1064	97.8	0.17
	M1	0	0	56	2		24	2.2	
Stage	I	25	30.1	1184	41.9	0.06	426	39.3	0.15
	II	44	53	1248	44.2		476	44	
	III	14	16.9	337	11.9		157	14.5	
	IV	0	0	56	2		24	2.2	
	Unknown	0		8			5		
HG	1 or 2	75	90.4	1438	54.9	<0.001	576	57.3	<0.001
	3	8	9.6	1183	45.1		429	42.7	
	Unknown			212			83		
ER	Positive	75	90.4	1825	64.4	<0.001	·	·	·
	Negative	8	9.6	1008	35.6		·	·	
PgR	Positive	59	71.1	1420	50.1	<0.001	·	·	·
	Negative	24	28.9	1413	49.9		·	·	
HER2	Negative	79	97.5	1586	74.6	<0.001	·	·	·
	Positive	2	2.5	540	25.4		·	·	
	Unknown *	2		707			·	·	
Subtype	Luminal A	74	91.4	1088	51.2	<0.001	·	·	·
	Non-LA	7	8.6	1038	48.8		·	·	
	Unknown	2		707			·	·	
p53	0 or 1+	74	93.7	2146	77.7	0.001	951	89.7	0.26
	2+ or 3+	5	6.3	616	22.3		109	10.3	
	Unknown	4		71			28		
Ki-67		10.3 ± 10.6		20.6 ± 19.8		<0.001	13.5 ± 13.2		0.03
Operation	BCS	57	68.7	2109	75.1	0.18	786	73	0.39
	Mastectomy	26	31.3	698	24.9		290	27	
	None	0	0	26			3		
Adjuvant therapy									
Chemotherapy	Yes	68	81.9	2383	84.1	0.59	922	84.7	0.49
	No	15	18.1	450	15.9		166	15.3	
Hormone therapy	Yes	79	95.2	2113	74.6	<0.001	1057	97.2	0.31
	No	4	4.8	720	25.4		31	2.8	
Anti-HER2 therapy	Yes^†^	1	1.2	164	5.8	0.08	·	·	·
	No	82	98.8	2669	94.2		·	·	
Radiotherapy	Yes	64	77.1	2323	82	0.25	879	80.8	0.41
	No	19	22.9	510	18		209	19.2	

* Including 2+ for HER2 by immunohistochemistry without FISH.

† Thirty-seven of these patients were enrolled in the Adjuvant Lapatinib and/or Trastuzumab Treatment Optimisation (ALTTO) trial [25] and 53 patients were enrolled in the Tykerb Evaluation After Chemotherapy (TEACH) trial [26].

BCS, breast-conserving surgery; ER, estrogen receptor; HER2, human epidermal growth factor receptor 2; HG, histologic grade; M, distant metastasis at diagnosis; pT, pathological tumor stage; pN, pathological nodal stage; PgR, progesterone receptor.

Compared to the IDC group, significantly more ILC patients presented with a low histologic grade (HG) (HG1 or 2, 90.4% vs. 54.9%, *P *< 0.001) and a large tumor size (≥T2, 59.8% vs. 38.8%, *P *= 0.001), although no difference was noted with respect to nodal involvement (43.9% vs. 41.3%, *P *= 0.18).

Significantly more tumors were positive for hormone receptors and had a negative HER2 status in the ILC group as compared to the IDC group (ER^+^, 90.4% vs. 64.4%, *P *< 0.001; PgR^+^, 71.1% vs. 50.1%, *P *< 0.001; HER2^-^, 97.5% vs. 74.6%, *P *< 0.001), with a greater proportion of the luminal A subtype in the ILC group (91.4% vs. 51.2%, *P *< 0.001). In addition, the mean Ki-67 value was lower in the ILC group compared to the IDC group (10.3 ± 10.6% vs. 20.6 ± 19.8%, *P *< 0.001).

Because 91.4% of ILC were of the luminal A subtype, we further compared ILC to the 1,088 LA-IDC. Whereas significant differences between ILC and LA-IDC were found for size, HG and Ki-67, the rates of nodal involvement and the expression of p53 were similar (Table 1).

The treatment modalities in the ILC group were also comparable to those used in the LA-IDC group. One of two patients with a HER2 positive tumor among the ILC group who developed disease recurrence received anti-HER2 treatment upon recurrence.

### Univariate analysis of DFS and OS of ILC compared to IDC patients

During the median follow-up of 56.4 (range 4.9-136.6) months, 365 patients experienced disease recurrence (6/83 ILC vs. 359/2833 IDC, *P *= 0.18) and 213 patients died (3/83 ILC vs. 210/2833 IDC, *P *= 0.28). One ILC patient experienced local recurrence, one contralateral breast cancer, and four distant metastasis.

Table 2 shows the results of the univariate analysis of DFS and OS of the ILC group and of all IDC patients. Significant prognostic factors for DFS were age at diagnosis; tumor size; lymph node involvement; individual ER, PgR, and HER2 statuses; p53 (0 or 1+ vs. 2+ or 3+); Ki-67 (cut-off: 20%); the type of operation (breast conserving surgery [BCS] vs. mastectomy); adjuvant hormone therapy; and intrinsic subtype (luminal A vs. non-luminal A). However, there was no significant difference in the 5-year DFS rate between ILC and all IDC (91.7% in ILC vs. 87.4% in IDC, *P *= 0.31).

**Table 2:** Univariate analysis of disease-free survival (DFS) and overall survival (OS) of all patients

Characteristic		Patients (n)	5-yr DFS rate (%)	*P*	5-yr OS rate (%)	*P*
Age	<35 yrs	226	79.3	<0.001	86.3	<0.001
	≥35 yrs	2690	88.2		93.1	
pT	T1	1763	91.9	<0.001	95.1	<0.001
	≥T2	1147	81		89.2	
	Unknown	6				
pN	Negative	1685	93.8	<0.001	95.9	<0.001
	Positive	1191	81.4		89.6	
	Unknown	40				
M	0	2860			93.9	<0.001
	1	56			24.6	
Stage	I	1209	95.2	<0.001	97.2	<0.001
	II	1292	89.4		94.8	
	III	351	69.2		80.4	
	IV	56			24.6	
	Unknown	8				
HG	1 or 2	1513	88.7	0.39	93	0.84
	3	1191	86.8		92.7	
	Unknown	212				
ER	Positive	1900	91.1	<0.001	95.7	<0.001
	Negative	1016	80.6		86.8	
PgR	Positive	1479	92.4	<0.001	96.6	<0.001
	Negative	1437	82.7		88.7	
HER2	Negative	1665	86.1	<0.001	91.4	0.001
	Positive	542	78.4		88.3	
	Unknown*	709				
Subtype	Luminal A (LA)	1162	89.3	<0.001	93.9	<0.001
	Non-LA	1045	78.5		87.1	
	Unknown	709				
Histological type	ILC	83	91.7	0.31	93.6	0.38
	IDC	2833	87.4		92.5	
p53	0 or 1+	2220	90.9	<0.001	95.2	<0.001
	2+ or 3+	621	81.8		87.1	
	Unknown	65				
Ki-67	≤20%	1902	90.1	<0.001	94.4	<0.001
	>20%	700	83.4		89.1	
	Unknown	314				
Operation	BCS	2166	89.5	<0.001	94.6	<0.001
	Mastectomy	724	84.2		88.6	
	None	27				
Adjuvant therapy						
Chemotherapy	Yes	2318	87.7	0.38	92.6	0.87
	No	556	86.6		92.5	
Hormone therapy	Yes	2192	90.4	<0.001	95.6	<0.001
	No	724	78.9		83.9	
Anti-HER2 therapy	Yes in HER2-positive ^†^	147	80.7	0.77	93.1	0.48
	No in HER2-positive	395	77.4		86.9	
	HER2-negative or unknown ^§^	2374				
Radiotherapy	Yes	2387	87.8	0.4	92.3	0.38
	No	529	86.3		92.9	

* Including 2+ for HER2 by immunohistochemistry without FISH.

† Thirty-seven of these patients were enrolled in the Adjuvant Lapatinib and/or Trastuzumab Treatment Optimisation (ALTTO) trial [25] and 53 patients were enrolled in the Tykerb Evaluation After Chemotherapy (TEACH) trial [26].

§ No anti-HER2 therapy due to negative or unknown for HER2 by immunohistochemistry without FISH.

ER, estrogen receptor; HER2, human epidermal growth factor receptor 2; HG, histologic grade; M, distant metastasis at diagnosis; pT, pathological tumor stage; pN, pathological nodal stage; PgR, progesterone receptor.

In the univariate analysis of OS, age, tumor size, nodal status, distant metastasis at diagnosis, individual ER, PgR, and HER2 statuses, p53, Ki-67, the type of operation, adjuvant hormone therapy and intrinsic subtype were prognostic factors. The 5-year OS rate was 93.6% for the ILC group and 92.5% for the IDC group (*P *= 0.38).

**Figure 2:**
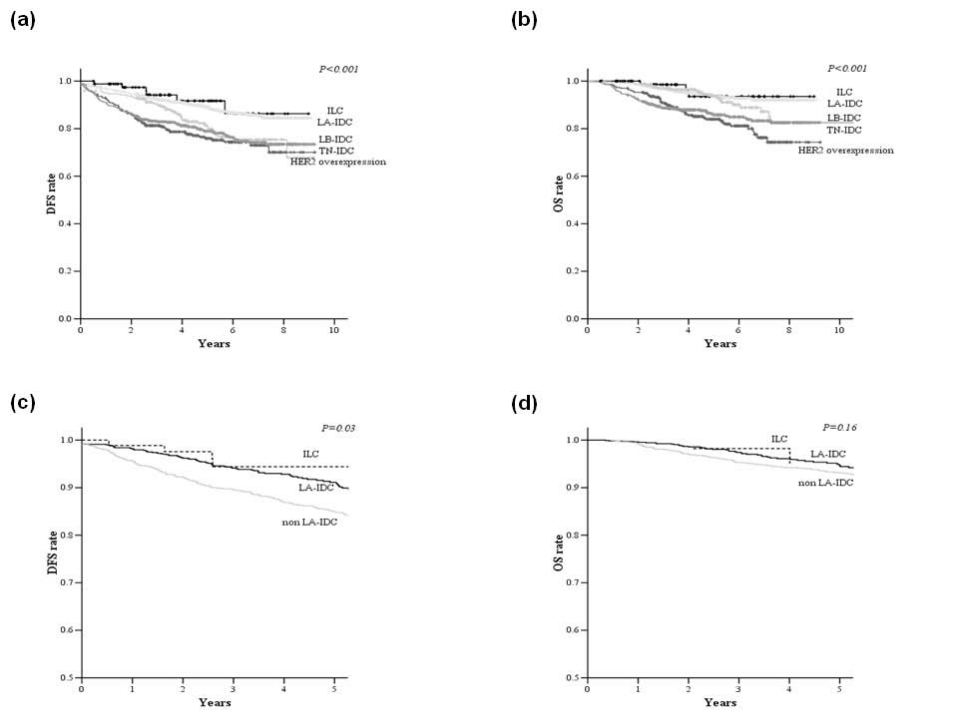
**Clinical outcomes according to subtypes**. Disease-free survival (DFS) curves (a) and overall survival (OS) curves (b) for patients with invasive lobular carcinoma (ILC) and 4 subtypes of invasive ductal carcinoma (IDC). DFS curves (c) and OS curves (d) for patients with ILC, LA and non-LA subtypes, adjusting for other prognostic factors in multivariate analysis. Abbreviations: ILC, invasive lobular carcinoma; LA-IDC, luminal A subtype of invasive ductal carcinoma; LB-IDC, luminal B subtype of invasive ductal carcinoma; TN-IDC, triple-negative subtype of invasive ductal carcinoma.

In this study, we classified the total 2,916 patients into ILC and four subtypes of IDC and compared the clinical outcomes. Figure 2 (a, b) presents the DFS and OS curves. The prognosis of ILC was similar to that of LA-IDC and was more favorable than with other subtypes of IDC; the 5-year DFS rates being 91.7% vs. 89.1% for LA-IDC, 80.7% for the luminal B subtype of IDC (LB-IDC), 78.9% for the triple-negative subtype of IDC (TN-IDC), and 75.9% for the HER2 overexpressing subtype (*P *< 0.001). The 5-year OS rates were 93.6%, vs. 93.4%, 92.8%, 85.8%, and 83.9%, respectively (*P *< 0.001).

### Multivariate analysis of DFS and OS of ILC compared to IDC, LA-IDC, and non-LA-IDC

Using the significant variables determined by the univariate analysis, we performed a multivariate analysis for DFS and OS (Table 3). Patients younger than 35 years (HR 2.17. 95% CI 1.55-3.02, *P *< 0.001), with a larger tumor size (HR 1.85, 95% CI 1.44-2.37, *P *< 0.001), and lymph node involvement (HR 2.81, 95% CI 2.15-3.68, *P *< 0.001) demonstrated an unfavorable prognosis (Table 3). Non-LA-IDC (LB-IDC, TN-IDC, and HER2-overexpressing subtypes) showed a more unfavorable prognosis compared to LA-IDC (HR 1.69, 95% CI 1.23-2.33, *P *= 0.001), but the DFS rates for ILC and LA-IDC were similar (HR 0.77, 95% CI 0.31-1.90, *P *= 0.57).

**Table 3:** Multivariate analysis of disease-free survival (DFS) and overall survival (OS)

	DFS			OS		
		
	HR	95% CI	*P*	HR	95% CI	*P*
Age (<35 yrs)	2.17	(1.55-3.02)	<0.001	1.96	(1.27-3.02)	0.002
pT (≥T2)	1.85	(1.44-2.37)	<0.001	1.72	(1.24-2.40)	0.001
pN (positive)	2.81	(2.15-3.68)	<0.001	2.61	(1.84-3.69)	<0.001
M (1)	·	·	·	10.75	(6.29-18.37)	<0.001
Subtype						
LA-IDC		1 (ref)			1 (ref)	
non LA-IDC	1.69	(1.23-2.33)	0.001	1.5	(0.97-2.33)	0.07
ILC	0.77	(0.31-1.90)	0.57	0.75	(0.18-3.09)	0.7
p53 (>25%)	1.27	(0.96-1.66)	0.09	1.64	(1.17-2.31)	0.004
Ki-67 (≥20%)	1.06	(0.81-1.39)	0.67	1.08	(0.77-1.50)	0.67
Operation (mastectomy)	1.15	(0.89-1.48)	0.29	1.14	(0.82-1.59)	0.44
Hormone therapy (no)	1.19	(0.88-1.62)	0.25	1.81	(1.21-2.71)	0.004

CI, confidence interval; HG, histologic grade; HR, hazard ratio; LA, luminal A; M, distant metastasis at diagnosis; pT, pathological tumor stage; pN, pathological nodal stage

For OS, a young age (HR 1.96. 95% CI 1.27-3.02, *P *= 0.002), larger tumor size (HR 1.72, 95% CI 1.24-2.40, *P *= 0.001), lymph node involvement (HR 2.61, 95% CI 1.84-3.69, *P *< 0.001), the presence of distant metastasis at first diagnosis (HR 10.75, 95% CI 6.29-18.37, *P *< 0.001), p53 overexpression (HR 1.64, 95% CI 1.17-2.31, *P *= 0.004), and no hormone therapy (HR 1.81, 95% CI 1.21-2.71, *P *= 0.004) were identified as independent factors that were significantly associated with mortality (Table 3). However, OS did not differ between ILC and LA-IDC patients (ILC; HR 0.75, 95% CI 1.18-3.09, *P *= 0.70).

Figure 2 (c, d) shows the DFS and OS curves adjusted for other prognostic factors for ILC and IDC subtypes. The DFS curves for ILC were similar to those for LA-IDC and were more favorable than those for non-LA-IDC (*P *= 0.03). The OS of ILC and LA-IDC was better than that of non-LA-IDC, but the difference did not reach statistical significance (*P *= 0.16).

## Discussion

ILC constitutes 2-4% of all breast cancer in Korea, as presented in the current study, which is much lower than the rate observed in most Western reports [1-3]. Although ILC occurs more often in older women in Western countries [9,10], our series demonstrated that age distributions were the same as those for overall IDC at diagnosis. Notably, the peak age of breast cancer patients in Korea is the late 40 s, which is 10 to 20 years younger than that in Western countries [4].

In the present study, the tumor size of ILC was larger than that of IDC, as observed in other studies [10,18,19]. Detection may be delayed because ILC is often clinically impalpable or mammographically invisible due to a lack of desmoplasia in the stroma [20]. Despite the larger tumor size, the rate of lymph node involvement in ILC did not differ from that in general IDC, which may reflect the slow growth rate of ILC, and this finding is consistent with other reports [10,18]. Due to the difficulty associated with early detection, the larger tumor size in ILC adversely affected the outcomes of patients with poor DFS in the present study. We reported that ILC had lower histologic grade than IDC. Previously, Li et al. analyzed Surveillance, Epidemiology, and End Results Program data and demonstrated that ILC showed lower tumor grade than IDC specifically in 30-49 years old patient group [21]. Although Rakha et al. reported that histologic grade of ILC provided a strong predictor of outcome in breast cancer patients and should be provided routinely in pathology reports, we did not find such correlation [22].

One of the objectives of the present study was to characterize more comprehensively the biological phenotype of ILC. As previously reported [6,10], the majority of ILC showed ER or PgR positivity and HER2 negativity, which we defined as consistent with the LA-IDC subtype. Weigelt et al. also showed that most ILC fall into luminal A molecular subtype, although some ILC had cluster with either HER2 subtype or apocrine subtype [23]. Furthermore, ILC demonstrated lower p53 and Ki-67 expression compared to IDC and LA-IDC. All of these characteristics suggest that ILC likely originates from more differentiated luminal cells [24].

Previous studies have reported that the prognosis of ILC patients is similar to that of IDC patients [6,10,18], as confirmed in the present study. Arpino et al. analyzed 4,140 ILC patients and 45,169 not otherwise specified IDC patients and reported that the histologic type did not affect the prognosis despite the favorable biological phenotype of ILC [10]. However, the outcome of ILC in the present study was comparable to that of LA-IDC and significantly better than the outcomes of other, non-LA-IDC subtypes, when we further analyzed the prognosis by breast cancer subtype.

This study demonstrated a new aspect of ILC after construing the data including biologic markers other than general tumor characteristics in the consecutive breast cancer patients who received consistent therapeutic approaches at a single center. Similar clinicopathological characteristics and clinical outcomes between ILC and LA-IDC were discovered after we further compared ILC with the four subtypes. To our knowledge, this is the first report to show such similarities between ILC and LA-IDC.

## Conclusions

ILC has distinct clinicopathological characteristics with a larger tumor at presentation, a lower HG, ER/PgR positive and HER2 negative status, and low Ki-67 expression, as compared to overall IDC. This study shows that most ILC are luminal A breast cancer, the prognosis of ILC is similar to that of LA-IDC, and both are better than the other subtypes.

## Abbreviations

CI: confidence interval; DFS: disease-free survival; ER: estrogen receptor; FISH: fluorescence in situ hybridization; HER2: human epidermal growth factor receptor-2; HR: hazard ratio; IDC: invasive ductal carcinoma; IHC: immunohistochemistry; ILC: invasive lobular carcinoma; LA: luminal A; OS: overall survival; PgR: progesterone receptor; TNBC: triple negative breast cancer

## Competing interests

The authors declare that they have no competing interests.

## Authors' contributions

SYJ participated in study design, data analysis and interpretation, manuscript drafting and revision. JJ participated in data acquisition. SHS participated in data acquisition. YK participated in data acquisition and manuscript revision. EAK participated in data acquisition and manuscript revision. KLK participated in data acquisition and manuscript revision. KHS participated in data acquisition and manuscript revision. KSL participated in data acquisition and manuscript revision. IHP participated in data acquisition and manuscript revision. SL participated in data acquisition and manuscript revision. SWK participated in data acquisition and manuscript revision. HSK participated in data acquisition and manuscript revision. JR participated in study design, data analysis and interpretation, manuscript drafting and revision, and study supervision. All authors read and approved the final manuscript.

## Pre-publication history

The pre-publication history for this paper can be accessed here:

http://www.biomedcentral.com/1471-2407/10/664/prepub
